# Pancreas β cell regeneration and type 1 diabetes (Review)

**DOI:** 10.3892/etm.2014.2163

**Published:** 2014-12-30

**Authors:** JINXIAO WU, XIYAN YANG, BIN CHEN, XIUPING XU

**Affiliations:** 1 Department of Endocrinology, Beijing Army General Hospital, Beijing 100700, P.R. China; 2 Department of Cardiology, Beijing Chaoyang Hospital, Capital Medical University, Beijing 100020, P.R. China

**Keywords:** diabetes mellitus, endocrine pancreas, β cell regeneration, type 1 diabetes

## Abstract

Diabetes mellitus, which may cause hyperglycemia and a number of complications, mostly results from a deficiency of β cell mass (type 1 diabetes) or a limitation of β cell function (type 2 diabetes). Currently, enhancing β cell regeneration and increasing cell proliferation have not only been described in experimental diabetes models, but have also been proven to improve outcomes for patients with diabetes. Therefore, understanding the mechanisms controlling the development and regeneration of β cells in the human pancreas may be helpful for the treatment of β cell-deficient disease. In this review, we first introduce the various cell types in the adult pancreas and thereby clarify their functions and origins. Then, the known mechanisms of β cell development and expansion in the normal human pancreas are described. The potential mechanisms of β cell regeneration, including β cell self-replication, neogenesis from non-β cell precursors and transdifferentiation from α cells, are discussed in the next part. Finally, the ability of the pancreas to regenerate mature β cells is explored in pathological conditions, including type 1 diabetes, chronic pancreatitis and persistent hyperinsulinemic hypoglycemia of infancy.

## 1. Introduction to cell types

The pancreas, a key regulator of nutrient digestion, absorption and utilization, is a mixed gland composed of two functionally and morphologically distinct tissues, with exocrine (99%) and endocrine (1%) components (1). The exocrine pancreas consists of acinar and ductal cells. Food ingestion triggers the nerve and hormonal responses in the intestine and induces the release of gastrin, secretin and cholecystokinin from the gastric G-cells, duodenal K-cells and duodenal L-cells, respectively. Then acinar cells are activated, which secrete multiple types of digestive enzymes, which are drained towards the duodenum by mature duct cells and directly participate in nutrient digestion (2,3).

The endocrine pancreas disperses in the exocrine parenchyma and is formed of islets of Langerhans. It consists of five cell types, namely α, β, δ, ɛ and pancreatic polypeptide (PP) cells (3). In rodents, 80% of islet cells are formed by β cells, which are considered as the only source of insulin (3–5). The other cells (α, δ, ɛ and PP cells) secrete glucagon, somatostatin, ghrelin and PP, respectively (4,5). Following food intake, the rising blood glucose levels cause β cells to produce insulin. The insulin represses glycogenolysis and neoglucogenesis, and thereby promotes glucose uptake by muscular or adipose tissue. However, when blood glucose is low, α cells secrete glucagon and stimulate hepatic glycogenolysis and neoglucogenesis, which causes glucose to return to its normal level. The function of somatostatin and PP is to negatively regulate the secretive ability of α, β and acinar cells. Ghrelin is able to repress β cell secretion. All of these hormones are involved in the regulation of nutrient metabolism and glucose homeostasis.

## 2. β cell development and expansion in normal pancreas

It is reported that the first clear morphological signs of the pancreas appear at approximately 8.5 days (6–8). In the mouse, the first insulin and glucagon-expressing cells may be observed at E9.5 and E10.5, respectively (9,10). However, these cells cannot be considered as mature endocrine pancreas, even if they produce insulin or glucagon. The initial pancreatic cells proliferate and peak at E13.5 (11). The multipotent progenitor cells express Ptf1a, c-myc and carboxypeptidaseA1 (CPA1) which are located at the distal tip of growing epithelium (12). These CPA1-positive cells have the ability to generate numerous types of pancreatic cells (12). Another significant molecular marker is Ngn3, which controls endocrine cell fate. Ngn3-labeled endocrine progenitors differentiate to endocrine hormone-producing cell subtypes (13). In the absence of Ngn3, endocrine cells fail to develop (14). At E14.5, CPA1-positive progenitors move towards acinar lineage. At the same time, maturing insulin- or glucagon-labeled endocrine cells and amylase-labeled acinar cells increase and accumulate. From E15.5 to birth, δ cells appear and begin to express somatostatin (12). Then PP cells emerge and thereby the mature islets of Langerhans are formed (11).

β cells arise from duct-associated precursor cells, and are detected in or near to the primitive duct epithelium (6). β cells in the fetal pancreas demonstrate a notable ability to proliferate. This contributes to the primary linear expansion of β cells until birth. Differentiation from ductal precursors also plays a significant role in β cell expansion during fetal life (6). In humans, the fetal pancreas cells secrete insulin and glucagon (6). During the postnatal period, β cells continue to proliferate, but this is accompanied by apoptosis (6,15,16). The apoptosis diminishes the number of developing β cells. It is still unclear whether ductal precursors participate in the expansion of β cells following birth. However, numerous insulin-positive cells, which are associated with ducts, have been observed in infants not only in the pancreas of persistent hyperinsulinemic hypoglycemia of infancy (PHHI) but also in the pancreas without metabolic problems (17). This fact indicates a possibility that ductal precursors are one of the sources of β cells in the postnatal period.

In summary, both differentiation from duct-associated precursors and self-proliferation contribute to the expansion of the β cell mass in the fetal period, as well as possibly in the postnatal period. Considering that multiple abilities in the fetal pattern could be reactivated under specific conditions in adult organisms, it is likely that proliferation and differentiation from precursor cells also participates in β cell neogenesis in the adult pancreas.

## 3. β cell self-proliferation

It is known that the self-proliferation of β cells is an essential process for islet expansions. A series of lineage-tracing analyses has established that self-replication or proliferation is a notable source of β cell regeneration during physiological conditions as well as 70% partial pancreatectomy (PPX) (18). Brennand *et al* constructed an *in vivo* model to confirm the location where the insulin-secreting cells participate in the replication-mediated expansion of β cell mass (19). In addition, a DNA analog-based lineage-tracing technique proved that only β cells proliferate for β cell regeneration during normal physiological conditions, following 50% PPX or treatment with Exendin-4 (20).

In adult mice, it has been proven that the ability of β cells to replicate is an age-dependent process, which means that this type of proliferation decreases with aging (21,22). However, under the conditions of hyperglycemia and hyperinsulinemia, the older mice demonstrated an increase in β cell proliferation (23). This fact suggested that adult β cells maintained the ability to replicate, and may be used to satisfy the increasing metabolic demand. However, due to the limitation on the detection of dynamic β cell proliferation, human studies have to be carried out based on immunohistochemical markers of β cells, including Ki-67, which is negative in adult β cells (24). Notably, multiple studies have demonstrated that this marker was positive in diseased pancreas as well as normal pancreas (25,26). In addition, patients with type 2 diabetes do not exhibit an increased rate of β cell proliferation (26). All of these results from research on humans may not be so persuasive compared with animal experiments.

β cell self-proliferation requires the regulation of multiple cell cycle molecules, including cyclin D2, Cdk4, E2F1 and MLL (27–33). Cyclin D2 knockout mice have smaller islets, reduced β cell mass and notably limited β cell proliferation ability (27,28). In humans, overexpression of Cdk4 also induces β cell replication (29,30). Ectopic expression of E2F1 and AKT increases the absolute cell numbers in primary β cells due to the promotion of β cell proliferation and inhibition of cell death (31). Another *in vivo* study of overexpression revealed that E2F1 indeed improved β cell proliferation, but this proliferation was not sufficient to support the expansion of β cell mass (31). Further research should be performed in this field. MLL is a type of trithorax TrxG protein. It has been proven that MLL was located at the p27kipl and p18Ink4c promoters and then modulated pancreatic islet growth (32,33).

In summary, β cells proliferate in the adult pancreas of humans and mice. This proliferation may be regulated by multiple cell cycle-related molecules. Although immunohistochemical experiments demonstrated a positive result, whether β cell proliferation is a major part of β cell regeneration in the adult human pancreas needs to be further studied and discussed.

## 4. Neogenesis from non-β cell precursors

The hypothesis is that injury (for example, inflammation) induces the activation of facultative precursor cells leading to the expansion of β cell mass (34,35). The differentiation of these precursors controls the function of the embryogenesis of endocrine pancreas (34,35). In adult mice, an experiment using partial pancreatic duct ligation proved the existence of endocrine progenitors, which can be labeled by a proendocrine factor Ngn3. These adult Ngn3-positive cells isolated from the adult pancreas included four major endocrine cell subtypes. Ngn3 knockout mice lose the ability to produce pancreatic endocrine cells (36).

The possibility of β cell neogenesis from non-β cell precursors was controversial until the appearance of lineage-tracing technology. A previous study revealed that β cells regenerate from ductal cell precursors in the mouse pancreas (37). In addition, neogenesis from ductal epithelium has been observed in the human pancreas based on the morphological structures of islet-ductal complexes (38). In these complexes, the endocrine pancreas appears to have an association with ductal structures. This phenomenon is observed in the human pancreas in chronic pancreatitis (CP) and asymptomatic pancreatic fibrosis in numerous clinical diagnoses (38). Although β cell neogenesis has not been proven by lineage-tracing experiments, fluorescence immunoassay of endocrine and ductal markers partly demonstrated the possibility of the presence of islet-duct complexes (39).

In addition to the ductal precursors, intra-islet precursor cells and acinar cells were also considered to participate in the neogenesis of β cells (35,40,41). In streptozotocin (STZ)-treated and normoglycemic by exogenous insulin (STZ/IN) mice and aging animals, there were two different β cell precursors in islets, which were characterized by Glut-2 and Pdx1/somatostatin, respectively (35). Although the details of these putative precursor cells remain unclear and controversial, certain researchers consider them to have a ductal origin (34,35).

In summary, in the adult injured pancreas, the duct epithelium often contains insulin-, glucagon- or Glut-2-expressing cells, which suggested the presence of islet neogenesis. Whether these insulin-positive cells in ductal structures are mature β cells remains to be confirmed; however, β cell neogenesis from non-β cell precursors (e.g. ductal precursors) may be a notable source of β cell regeneration in the human pancreas. In addition, these ductal progenitors may differentiate to hormone-producing cells, leading to the expansion of β cells or adjacence between new islets and the duct epithelium.

## 5. β cell formation from transdifferentiation of α cells

Previous studies have revealed that α cells, another endocrine cell type in the pancreas, may be a source of β cells in the adult mouse model (42,43). This phenomenon has not yet been reported in the human pancreas. However, researchers have observed that certain pancreatic cells exhibited double positivity of insulin and glucagon (16). In CP, α cells increased due to the neogenesis from ductal cells. These newly formed α cells have the potential to transdifferentiate to β cells. This phenomenon gained more attention since α cells are able to resist immune-mediated destruction in autoimmune diabetes (42–44).

Conditional expression of Pax4 initiates the conversion of α cells to functional β cells by activating certain transcription factors (45). Glucagon shortage induced the neogenesis of glucagon-producing cells. This phenomenon has also been observed in a glucagon receptor knockout mouse model, which demonstrated α cell hyperplasia (46). These α cells, originating from the reactivation of Ngn3, converted to a β cell phenotype and thereby produced insulin (45,47). The constitutive activation of Cdk4 was observed to increase the proliferation of β cells as well as ductal cells (48). Adult mice expressing the human diphtheria toxin receptor also demonstrated a regeneration of β cells through spontaneous conversion of α cells (42).

In summary, although further research needs to be carried out, the possibility that α cells transform to insulin-producing cells may provide one source of β cell regeneration in autoimmune-associated diabetes (type 1 diabetes), CP and pancreatic fibrosis.

## 6. β cell regeneration in pathological conditions

### Type 1 diabetes

Type 1 diabetes is a chronic autoimmune disease in which β cells are destroyed by autoimmune reactions (49). Prior to a discussion on β cell regeneration in type 1 diabetes, the question of whether the pancreas still has the ability to retain β cells following β cell destruction should be addressed. A number of clinical studies have observed that most patients with type 1 diabetes had residual β cells (50). In addition, a Juvenile Diabetes Research Foundation-funded network for pancreatic organ donors initiative has been established for a number of long-standing childhood onset diabetic organ donors (51). This suggests that the pancreas of patients still had residual β cells even with long-standing type 1 diabetes. Previous studies revealed that these residual β cells demonstrated heterogeneity in type 1 diabetes, and could be divided into two patterns, pattern A and B (16,52). Pattern A has almost no insulin-producing cells but has other endocrine pancreatic cells. Patients with pattern A do not exhibit residual β cells, but have another type of β cells, which are different from normal β cells due to their higher expression of Class I human leukocyte antigen and survivin. Pattern B has residual β cells. However, these residual β cells may not be associated with β cell regeneration. The possible reasons can be inferred as follows: i) All β cells are killed following the onset of disease, but certain partial β cells regenerate and restore fewer β cells. ii) The disease kills most but not all β cells. Further research should be performed to clarify how these regenerated β cells arrive in the pancreas and how they function.

## 7. Chronic pancreatitis (CP) and pancreatic fibrosis

CP is a progressive and destructive inflammatory process of multifactorial etiology that leads to irreversible obliteration of pancreatic tissue (53). In animal models, a number of studies have demonstrated the potential of pancreatic β cell regeneration following injury. Islet neogenesis is a possible mechanism for normal islet growth and regeneration following birth. Ductal-islet complexes and insulin-glucagon double-positive cells are observed in the adult pancreas with either CP or pancreatic fibrosis (38). This fact indicates the possibility of the presence of β cell regeneration in inflammatory pancreatic disease. Another study identified pancreatic ductal precursors as one source of islet neogenesis in human patients (39). Although the underlying mechanisms still need to be further explored, the studies above provide direct evidence of β cell regeneration in the human adult pancreas.

Extensive stellate cell-mediated fibrosis is a main characteristic of CP as well as pancreatic cancer (53,54). It was observed that bone marrow-derived stem cells could incorporate into the pancreas and differentiate to the exocrine compartment (55). This differentiation adopted various phenotypes depending on whether cells are recruited to an inflammatory or a carcinogenic pancreas. It is reported that autologous islet cell transplantation following total pancreatectomy for the treatment of CP with severe abdominal pain is the standard therapy (56). Moreover, Yatagai *et al* (57) reported that fibrosis exhibited insulin-glucagon double-positive cells, and proposed that fibrotic pancreas may be a potential source of β cells in the adult pancreas.

## 8. Persistent hyperinsulinemic hypoglycemia of infancy (PHHI)

PHHI is another pathological condition with evidence of β cell regeneration in the human pancreas. In PHHI, the majority of patients exhibit β cell abnormalities, which are associated with genetic mutations regulating β cell function. In a third of these patients, β cell hypertrophy may be observed (58,59). These endocrine cells appear as islet-like structures and are separated by acinar cells or other connective tissues (58,59). It is also reported that the proliferation of the endocrine cells is notably increased. In these islet-like structures, the percentage of β cells is 70–90%, which is much higher than that in normal tissue (60,61). The increased number of β cells makes certain islets larger than normal. Thus, β cell hyperplasia in adults contributes to PHHI. The main pathogenetic mechanism of endocrine cell hyperplasia is possibly the increased endocrine cell neogenesis rather than existing cell proliferation.

## 9. Conclusion

In this review, the mechanisms of β cell regeneration in physiological and pathological conditions are summarized, and the main factors regulating the regenerating processes are synopsized. Even in autoimmune-associated diabetes (type 1 diabetes) and inflammatory injury (CP and pancreatic fibrosis) the pancreas retains the potential of β cell regeneration. Future efforts should focus on the identification of underlying mechanisms of β cell regeneration in the human pancreas.
